# Immunology of cord blood T-cells favors augmented disease response during clinical pediatric stem cell transplantation for acute leukemia

**DOI:** 10.3389/fped.2023.1232281

**Published:** 2023-09-13

**Authors:** Roisin Borrill, Kay Poulton, Robert Wynn

**Affiliations:** ^1^ Blood and Marrow Transplant Unit, Royal Manchester Children’s Hospital, Manchester University NHS Foundation Trust, Manchester, United Kingdom; ^2^ Division of Infection, Immunity and Respiratory Medicine, Faculty of Biology, School of Biological Sciences, Lydia Becker Institute of Immunology and Inflammation, Medicine and Health, University of Manchester, Manchester, United Kingdom; ^3^ Transplantation Laboratory, Manchester University NHS Foundation Trust, Manchester, United Kingdom; ^4^ Manchester Academic Health Science Centre, University of Manchester, Manchester, United Kingdom

**Keywords:** cord blood, cord blood transplantation, acute leukemia, graft-vs.-leukemia, pediatric hematology, T-cells

## Abstract

Allogeneic hematopoietic stem cell transplantation (HSCT) has been an important and efficacious treatment for acute leukemia in children for over 60 years. It works primarily through the graft-vs.-leukemia (GVL) effect, in which donor T-cells and other immune cells act to eliminate residual leukemia. Cord blood is an alternative source of stem cells for transplantation, with distinct biological and immunological characteristics. Retrospective clinical studies report superior relapse rates with cord blood transplantation (CBT), when compared to other stem cell sources, particularly for patients with high-risk leukemia. Xenograft models also support the superiority of cord blood T-cells in eradicating malignancy, when compared to those derived from peripheral blood. Conversely, CBT has historically been associated with an increased risk of transplant-related mortality (TRM) and morbidity, particularly from infection. Here we discuss clinical aspects of CBT, the unique immunology of cord blood T-cells, their role in the GVL effect and future methods to maximize their utility in cellular therapies for leukemia, honing and harnessing their antitumor properties whilst managing the risks of TRM.

## Introduction

For children with high-risk acute leukemia, allogeneic HSCT is an important and effective treatment strategy. HSCT produces potent anti-leukemia activity through the dose intensification of chemotherapy used in conditioning and more importantly, the GVL effect mediated primarily through donor T-cells and other immune cells. Maximizing GVL must occur in balance with prevention of graft-vs.-host disease (GVHD), in which alloimmune T-cell responses are directed against healthy tissues resulting in patient morbidity and mortality in both its acute and chronic forms (1). Conventional HSCT includes bone marrow transplantation (BMT) and peripheral blood stem cell transplantation (PBSCT), often from family or matched unrelated donors (MUD). Cord blood has been used as a source of hematopoietic stem cells (HSCs) in transplantation for acute leukemia for over three decades (2). It has many important differences and consequent advantages compared to conventional stem cell sources, including significantly reduced relapse rates in acute leukemia, particularly for those patients with residual disease pre-transplant, and lower rates of chronic GVHD (3, 4). Here we discuss the key clinical and immunological features of cord blood, and in particular cord blood T-cells, in order to shed light on the mechanisms through which this augmented disease response occurs.

## Graft-vs.-Leukemia

T-cells are important mediators of GVL (5). In allogeneic HSCT, T-cell depletion with *ex vivo* graft manipulation or *in vivo* with T-cell depleting antibodies, such as anti-thymocyte globulin (ATG) or alemtuzumab, is used to prevent of GVHD. In malignancy, however, the use of T-cell depletion results in an increased risk of relapse (6–9). Conversely, relapse is reduced in those patients who develop GVHD and when relapse occurs, remission can be restored using donor lymphocyte infusions (DLI) (10–15). These clinical observations provide clear evidence for the potent role of T-cells in the GVL effect. In recent years, advancements in adoptive T-cell therapies, particularly chimeric antigen receptor (CAR) T-cells, have allowed sustained remission and cure to be attained in patients with previously incurable hematological malignancies, which further emphasizes the importance of T-cells in controlling malignant disease (16, 17).

T-cells recognize leukemia through interactions between their T-cell receptor (TCR) and human leukocyte antigen (HLA) molecules expressed on the surface of leukemia cells, which present antigenic peptide. HLA molecules are encoded by the human major histocompatibility (MHC) complex, which is a highly polymorphic region of genes located on chromosome 6 (18). CD8^+^ T-cells recognize peptide bound to HLA class I molecules, which are expressed on all nucleated cells, whereas CD4^+^ T-cells recognize HLA class II molecules, which are primarily expressed on specialized antigen-presenting cells (APCs). Both CD8^+^ and CD4^+^ T-cells mediate GVL reactions through interactions with antigen presented by HLA class I and class II molecules respectively (19).

HLA offers a potential important GVL target. In CBT, HLA-mismatch has shown to correlate with reduced relapse rates, with higher relapse rates following transplant in patients receiving the best matched cord blood units (20–22). Following haploidentical HSCT, loss of the entire mismatched haplotype through uniparental disomy of chromosome 6 p has been described in multiple patients (23). Leukemia relapse occurs as a consequence of this genetic event due to immune evasion. This suggests that in both the CBT and haplo-setting, mismatched HLA is a key target for GVL mediators. Loss of class I HLA expression through focal genetic deletions has also been described following matched allogeneic HSCT (24). In this setting, loss of HLA is likely to prevent presentation of important peptides involved in the GVL effect, for example, minor histocompatibility antigens (miHAs). These peptides are presented by major HLA molecules and differ between donor and recipient due to genetic polymorphisms meaning they can be recognized by engrafting T-cells and used to elicit a GVL effect (25, 26). Leukemia-associated antigens, expressed solely on leukemic cells and not normal tissues, are another potential target for GVL. There has been much work looking at identifying such antigens in order to generate effective, specific antigen-directed immunotherapy (27). Early clinical studies demonstrating that recipients of syngeneic HSCT have a higher incidence of relapse compared to allogeneic HSCT, suggest that it is not solely leukemia-associated antigens involved in the GVL effect, however, and a difference between donor and recipient is required (28). In double CBT (dCBT), in which two cord blood units are infused simultaneously in order to overcome limitations of cell dose, one unit asserts dominance over the other to become the engrafting unit. Higher CD3^+^ and naïve CD8^+^ T-cell content of the unit is positively associated with unit dominance in which one rejects the other (29). NK cell alloreactivity is also important in GVL, eliminating leukemia whilst protecting against GVHD (30). Following allogeneic transplant, development of antibodies against specific miHAs is positively correlated with survival, indicating a role of B-cells in the GVL effect also (31).

## Cord blood transplantation

The first successful CBT was performed in 1988 for a patient with a diagnosis of Fanconi's anemia (32). Today, there are cord blood banks situated in many countries across the globe with over 800,000 estimated cord blood units stored worldwide in public banks and over 4 million privately stored (33). It is feasible and practicable to collect and store cord blood stem cells through cryopreservation without deleterious effect on their viability (2). This means that cord blood has the advantage of being readily available without the need for stem cell harvest from an adult or sibling donor, which can dramatically reduce donor search time and procurement of stem cells for transplant from 2 to 4 months for bone marrow (BM) or peripheral blood stem cells (PBSCs) to as little as 2 weeks for cord blood (34, 35). This can have important clinical consequences for those patients with high-risk malignancies. In addition to this, cord blood registries are able to provide donors from a larger selection of ethnic backgrounds, which allows those groups who may have previously struggled to find an appropriately matched unrelated donor to be eligible for transplant (1). Despite this, reported rates of cord blood use for allogeneic transplant have been steadily declining with the rise of haploidentical transplants as an alternative option for those without a related or unrelated fully matched donor (33, 36, 37).

## Outcomes after cord blood transplantation for acute leukemia

The greatest risk to survival in acute leukemia is relapse. In those patients with residual disease pre-transplant and highest disease risk, use of CBT reduces relapse rates compared to other stem cell sources (3, 4). Superiority of CBT to other cell sources in preventing relapse has been replicated in multiple studies in both adults and children (3, 4, 38–40). In 2007, Eapen et al. showed in a retrospective, registry study, that in children with acute leukemia there was significantly reduced relapse risk following a 5/8 HLA-mismatched CBT compared to BMT (relative risk 0.54; *p* = 0.0045) (20). Milano et al., reported in 2016, significant superiority of CBT in preventing relapse when compared to MUD and mismatched unrelated donor (MMUD) stem cell sources, in adults with acute leukemia and residual disease pre-transplant, (hazard ratio in the HLA-mismatched group, 3.01; *p* = 0.02 and hazard ratio in the HLA-matched group, 2.92; *p* = 0.007) (4). This translated into improved overall survival for CBT recipients with a higher risk of death in the MUD and MMUD groups (hazard ratio in the HLA-mismatched group, 2.92; *p* = 0.001 and hazard ratio in the HLA-matched group, 1.69; *p* = 0.08) (4). For those patients without residual disease the benefit from CBT was less evident with lower hazard ratios (hazard ratio in the HLA-mismatched group, 1.36; *p* = 0.3 and hazard ratio in the HLA-matched group, 0.78; *p* = 0.33). Ando et al. showed improved 2-year overall survival with CBT compared to BMT and PBSCT, in adults with acute leukemia regardless of disease status pre-transplant (76.4% vs. 62% and 67.2%; *p* = 0.021) (40). In a large, multi-center, retrospective review of pediatric patients with acute myeloid leukemia (AML), Horgan et al. reported dramatically reduced incidence of relapse at 2-years with CBT in comparison to other cell sources in those patients with detectable MRD (36.2% vs. 66.2%; hazard ratio 0.46; *p* = 0.007). This promoted improved disease-free survival in the cord blood group (50% vs. 21%; hazard ratio 0.55; *p* = 0.017) (3). Excellent outcomes have also been reported by Barker et al., in adults using dCBT with 3-year OS and progression-free survival (PFS) at 82% and 76% respectively (41). These results taken together indicate that CBT produces a significant GVL effect, which translates into better relapse and leukemia-free survival outcomes for patients, particularly in those with high-risk disease.

## Transplant-related mortality including infectious complications

Historically, CBT has been associated with a higher incidence of TRM and infectious complications in conjunction with delayed neutrophil and platelet engraftment (8, 20). Mortality during CBT in children has reduced over time (42). Early registry-based studies performed by Eapen et al. in both children and adults, described an increased risk of TRM using CBT compared to BM grafts (20, 43). For children receiving CBT with two-allelic mismatches the risk of TRM was greater than matched BM (relative risk 2.31; *p* = 0.003) and this risk was also evident with one-allelic mismatch (relative risk 1.88; *p* = 0.0455) (20). In adults receiving 4–6/6 CBT there was also increased TRM seen in comparison to fully-matched BM (relative risk 1.69; *p* < 0.01) and PBSC (relative risk 1.62; *p* < 0.01) (43). In comparison to 1 allele mismatched BM and PBSC grafts TRM was similar. In another study from 2001, Rocha et al. reported increased early TRM within the first 100 days post-transplant in patients receiving CBT with ATG serotherapy compared to BMT (hazard ratio 2.13; *p* < 0.001) (8). Increased incidence of early TRM with CBT has also been described by Weisdorf et al. within the first 3-months post-transplant (hazard ratio 2.83; *p* < 0.0001) but beyond 3-months TRM rates were similar between CBT and BM groups (hazard ratio 1.00; *p* = 0.99) (44). Konuma et al. examined outcomes of patients aged 55 and above receiving CBT without serotherapy against BM and PBSC recipients, finding reduced rates of TRM in the latter groups (hazard ratio 0.61; *p* < 0.001 and 0.63; *p* < 0.001 respectively). This has also been reported in children receiving T-replete CBT for myeloid malignancy with an increased rate of TRM compared to a comparator arm (hazard ratio 2.04; *p* = 0.042) (3). Other studies, however, have produced conflicting data with no significant increase in TRM detected with CBT (38, 40, 45–48). Heterogeneity of patient populations, disease groups, conditioning regimens, particularly the use of T-depleting serotherapy and outcome measures confounds direct comparison of these studies. TRM is a significant consideration in CBT and needs to be considered on the basis of risk to the individual patient and the risk of their disease.

The incidence of primary graft failure following CBT is around 11%–12% in adults and children, compared to 5%–6% with HLA-matched BM and PBSC grafts (49, 50). The risk of graft failure is lower in those patients undergoing transplantation for hematological malignancy (50). In CBT, for deaths primarily attributed to graft failure the TNC dose received is predictive (RR 0.4 for increasing dose; *p* < 0.001) but level of HLA-mismatch is not (51). Fully-HLA matched CBT is associated with improved neutrophil engraftment (relative risk 1.8; *p* < 0.001) although there is no significant differences between recipients of grafts with 1- or 2-HLA mismatches (relative risk 1.0; *p* = 0.896). Patients can be successfully salvaged following graft failure with a second allogeneic HSCT (49, 52, 53).

Infection is a large contributor to non-relapse mortality in all HSCTs including CBT. Viruses in particular can create a higher burden of morbidity and mortality in CBT (54). Members of the herpes virus family including cytomegalovirus (CMV), varicella zoster virus (VZV), human herpes virus 6 (HHV6) have been shown to have higher incidence with CBT (55–57). There are low rates of viral transmission with CBT due to screening, therefore this represents reactivation rather than primary infection. CMV can require longer duration of treatment with CBT (57). Risks from viruses can be mitigated, however, through routine testing, prophylaxis and pre-emptive treatment upon identification and this is an evolving area with improvements in supportive care and new therapies (58, 59). For example use of letermovir for CMV reactivation, has shown promising results that may translate into better outcomes in pediatric CBT in the future (59). The use of anti-thymocyte globulin (ATG) serotherapy can negatively impact infection rates in CBT and development of T-cell responses to common viral and bacterial pathogens (60, 61). In one study, ATG use in CBT was significantly associated with increased risk of CMV, EBV and adenovirus viraemia and death from viral infections (61). This occurred in conjunction with delayed immune reconstitution. Omitting T-cell depleting serotherapy in malignancy, may therefore improve both relapse and non-relapse mortality (62, 63). In adults, particularly elderly patients or those with multiple co-morbidities, reduced intensity conditioning (RIC) regimens used in conjunction with CBT can allow the graft itself to drive engraftment and GVL (64–66). Overall survival in these patients is comparable to those receiving myeloablative conditioning (MAC) regimens, although reduced non-relapse mortality is offset by increased risk of relapse in some studies (64). Other complications associated with CBT include autoimmune cytopenia and gastrointestinal complications including the cord colitis phenomenon (67, 68).

## GVHD and tolerance of HLA-mismatch

One advantage of cord blood is its greater tolerance of HLA-mismatch. Outcomes in children with acute leukemia following CBT with mismatch at one or two alleles are at least equivalent to fully matched unrelated donor transplants despite HLA disparity (20, 45). The important composite end point of GVHD-free, relapse-free survival is superior in CBT recipients compared to other stem cell sources (3, 39). Across all HSCTs, the impact of HLA-mismatch on survival also appears to be less pronounced if there is a higher risk of the disease (69).

HLA-mismatch can result in better outcomes when transplanting for malignancy. Use of HLA-mismatch in CBT correlates with reduced relapse risk in both children and adults (20, 21). Sanz et al. demonstrated in adults with AML, that an HLA-mismatch of 2 or more alleles was associated with a lower 5-year incidence of relapse (without an increase in TRM Mismatch ≥2 22%; mismatch <2 44%; *p* = 0.04) (21). Yokoyama et al. further examined the relationship between HLA-mismatch and outcomes in CBT for acute leukemia, and reported inferior survival only in children receiving CBT with 4 or more allelic mismatches (hazard ratio 2.03; *p* = 0.011) (22). Overall survival was comparable between all other groups. In addition to this, a significantly higher incidence of relapse was noted in adults who had received a fully HLA-matched 8/8 CBT (hazard ratio 1.53; *p* = 0.0037). Altogether these are important observations that greater HLA-mismatch can be tolerated by CBT and that this could mediate greater GVL effect without excessive TRM.

The incidence of grade II-IV acute GVHD is higher in CBT than for MSD transplants, reported at around 35%–50% (40, 44, 45, 70). Although there is variation within the literature, the risk of grade II-IV acute GVHD is similar between CBT and MUD transplants if bone marrow is used as the cell source, but lower in CBT if PBSCs are utilized (20, 40, 44). CBT confers lower risk of acute GVHD than MMUD transplants (39, 43). There is reduced risk of severe grade III-IV acute GVHD with CBT compared to both MUD and MMUD transplants (39, 40, 48). Despite the high incidence of acute GVHD with CBT this does not translate into increased rates of chronic GVHD. The risk of developing chronic GVHD is lower in CBT than both MUD and MMUD transplants using both BM and PBSC cell sources (39, 40, 43–45, 70). The incidence of chronic GVHD using CBT is between 5%–28%, compared to 44%–53% for MUD transplants (3, 40, 44). Haploidentical transplantation is another alternative donor stem cell source option, often considered in place of CBT. Lower chronic GVHD rates are seen consistently with CBT in comparison to haploidentical transplantation (71, 72). Table 1 summarizes the literature on acute and chronic GVHD based on donor type and cell source.

**Table 1:** Summary of acute and chronic GVHD by donor type and cell source.

Paper	Patient group	Donor and cell source	Acute GVHD	Chronic GVHD
Rocha et al. (8)	541 children ALL, AML	CBT vs. MUD (BMT)	Grade II-IV lower with CBT HR 0.5 (*p* = 0.001)	Lower in CBT HR 0.24 (*p* = 0.002)
Eapen et al. (20)	785 children ALL, AML	CBT vs. MUD (BMT)	Grade II-IV aGVHD similar risk across groups 8/8 CBT vs. MUD HR 0.45 (*p* = 0.0387) 2-allele mismatched CBT vs. MUD HR 0.92 (*p* = 0.6521)	Similar risk across groups 8/8 CBT vs. MUD HR 0.76 (*p* = 0.4774) 2-allele mismatched CBT vs. MUD HR 0.72 (*p* = 0.1615)
Takahashi et al. (48)	171 adults ALL, AML, CL, lymphoma	CBT vs. MRD or MMRD (BMT or PBSCT)	Grade III-IV aGVHD risk lower in CBT HR 0.38 (*p* = 0.04)	Extensive cGVHD risk lower in CBT HR 0.49 (*p* = 0.01)
Eapen et al. (43)	1,525 adults ALL, AML	CBT vs. MUD or MMUD (BMT or PBSCT)	Grade II-IV aGVHD risk lower in CBT than 7/8 BMT HR 0.59 (*p* = −.01); 8/8 PBSC HR 0.59 (*p* < 0.01); 7/8 PBSC HR 0.49 (*p* < 0.01)	Lower risk in CBT than all groups; vs. 8/8 BMT HR 0.63 (*p* = 0.01); 7/8 BMT HR 0.59 (*p* = 0.01); 8/8 PBSCT HR 0.38 (*p* < 0.01); 7/8 PBSCT HR 0.46 (*p* < 0.0001)
Weisdorf et al. (44)	740 adults AML	CBT vs. MUD or MMUD (BMT or PBSCT)	Similar incidence across groups CBT 35% vs. MUD 36% (*p* = 0.69) and MMUD 44% (*p* = 0.14)	3-year incidence lower in CBT 28% vs. MUD 53% (*p* < 0.0001) and MMUD 59% (*p* < 0.0001)
Ruggeri et al. (72)	1,446 adults ALL, AML	CBT vs. haplo (BMT or PBSCT)	Similar incidence of grade II-IV aGVHD CBT 31% vs. haplo 27% (*p* = 0.1) in AML and CBT 33% vs. haplo 31% (*p* = 0.71) in ALL	Lower risk of cGVHD in CBT vs. haplo for AML HR 0.63 (*p* = 0.008) and ALL HR 0.58 (*p* = 0.01)
Konuma et al. (70)	2,091 adults ALL, AML	CBT vs. MSD (BMT or PBSCT)	Grade II-IV aGVHD risk lower in BMT HR 0.53 (*p* < 0.001) and PBSC HR 0.64 (*p* < 0.001) than CBT	Higher risk in PSBCT HR 1.76 (*p* < 0.001) and trend towards higher risk in BMT HR 1.33 (*p* = 0.06) than CBT
Keating et al. (45)	317 children AML	CBT vs. MSD vs. MUD (BMT or PBSCT)	Grade II-IV aGVHD incidence higher in CBT 52% vs. MSD 25% and MUD 43% (*p* < 0.01)	Lower incidence in CBT 21% vs. MUD 48%
Mehta et al. (39)	1,613 children ALL, AML	CBT vs. MMUD (BMT)	Lower incidence of grade III-IV aGVHD in CBT 18% vs. 7/8 BM 29% (*p* < 0.001) Increased risk of grade III-IV aGVHD with 7/8 BM HR 1.7 (*p* = 0.006)	Lower estimated 1-year incidence cGVHD in CBT 22% vs. 7/8 BMT 28% (*p* = 0.004)
Ando et al. (40)	310 adults ALL, AML, CML	CBT vs. MSD or MUD (BMT or PBSCT)	Similar incidence grade II-IV aGVHD in CBT 40.4% vs. BM 54.2% and PBSC 45.2% (*p* = 0.201) Lower incidence of grade III-IV aGVHD in CBT 5.2% vs. BM 11.6% and PBSC 16.6% (*p* = 0.047)	Lower incidence of cGVHD in CBT 27.5% vs. BM 43.1% and PBSCT 44.5% (*p* = 0.025)
Konuma et al. (71)	1,313 adults AML	CBT vs. haplo (BMT or PBSCT)	Higher incidence of grade II-IV aGVHD in CBT 39% vs. haplo 30% (*p* = 0.013)	Lower incidence of cGVHD in CBT 13% vs. haplo 22% (*p* = 0.006)
Wagner et al. (73)	708 adults and children ALL, AML	CBT vs. haplo (BM Tor PBSCT)	Higher incidence of grade II-IV aGVHD in CBT 48% vs. haplo 26% (*p* < 0.001)	Similar incidence of cGVHD in CBT 43% vs. haplo 38% (*p* = 0.42)
Horgan et al. (3)	367 children AML	CBT vs. MSD, MUD, haplo comparator arm (BMT or PBSCT)	Not reported	Lower incidence cGVHD in CBT 5% vs. comparator arm 19.4% Lower risk of cGVHD in CBT HR 0.25 (*p* = 0.003)

aGVHD, acute graft-versus-host disease; ALL, acute lymhoblastic leukemia; AML, acute myeloid leukemia; BMT, bone marrow transplant; CBT, cord blood transplant; cGVHD, chronic graft-versus-host disease; CL, chronic leukemia; CML, chronic myeloid leukemia; GVHD, graft-versus-hist disease; haplo, haploidentical transplant; HR, hazard ratio; MMRD, mismatched related donor; MMUD, mismatched unrelated donor; MSD, matched sibling donor; MRD, matched related donor; MUD, matched unrelated donor; PBSCT, peripheral blood stem cell transplant.

There is increased risk of development of chronic GVHD in adults compared to children receiving CBT (relative risk 5.7; *p* < 0.05) as well as BMT (relative risk 4.8; *p* < 0.05) and PBSCT (relative risk 10.0; *p* < 0.05) (74, 75). For children receiving MSD BMT, there is reduced incidence of grade II-IV acute and chronic GVHD in younger patients aged 2–12 years, than those older patients aged 13–18 years (76). This is not seen in CBT with similar rates of acute and chronic GVHD across all age groups of children (77).

It is important to consider when comparing CBT with MSD or MUD transplants that CBT will be HLA-matched at 5 or more alleles, rather than fully HLA-matched at 8 or 10 alleles as when using adult donors, and therefore more GVHD may be expected. In addition to this, the presence of GVHD is associated with reduced risk of leukemia relapse due to the corresponding GVL effect (13, 14). Conversely, the management of severe grade III-IV acute GVHD includes corticosteroids and other immunosuppressive agents (78). Prolonged use of immune suppression may contribute to increased risk of relapse, and its rapid withdrawal can be effective in the management of early relapse (78–80). GVHD itself can also negatively impact immune reconstitution, particularly thymopoiesis (81). Naïve T-cells induce potent alloreactive responses in xenograft studies, and there is clinical data to suggest that graft depletion of naïve T-cells reduces the severity of GVHD (82, 83). A peak of activated CD8^+^ T-cells expressing activation marker CD38 in peripheral blood has been associated with development of acute GVHD (84). This implies that high numbers of naïve T-cells transferred in cord blood grafts, differentiating into effectors in response to alloantigen could drive GVHD (85).

Research in the field of GVHD biomarkers is rapidly developing, to inform both diagnosis and prognosis of the condition (86). ST2 is one such biomarker that has been associated with development of acute GVHD after day 28 in CBT (87). Analysis of cell-signaling in the pathophysiology of GVHD has highlighted the importance of the rat sarcoma/ mitogen-activated protein kinase kinase/ extracellular receptor kinase (RAS/MEK/ERK) pathways in alloreactive T-cells. Detection of higher levels of phosphorylation of the ERK1/2 pathway in CD4^+^ T-cells has been described as a biomarker for the development of acute GVHD (88), MEK inhibitors have additionally been shown to preferentially inhibit cytokine production in naïve alloreactive T-cells (89). In the future, stratification of patients on the basis of biomarkers could facilitate decisions around therapeutic interventions for GVHD, allowing GVL to be maximized in those at low risk but earlier intervention in those at high risk of severe GVHD, reducing the burden of TRM in CBT.

**Figure 1:**
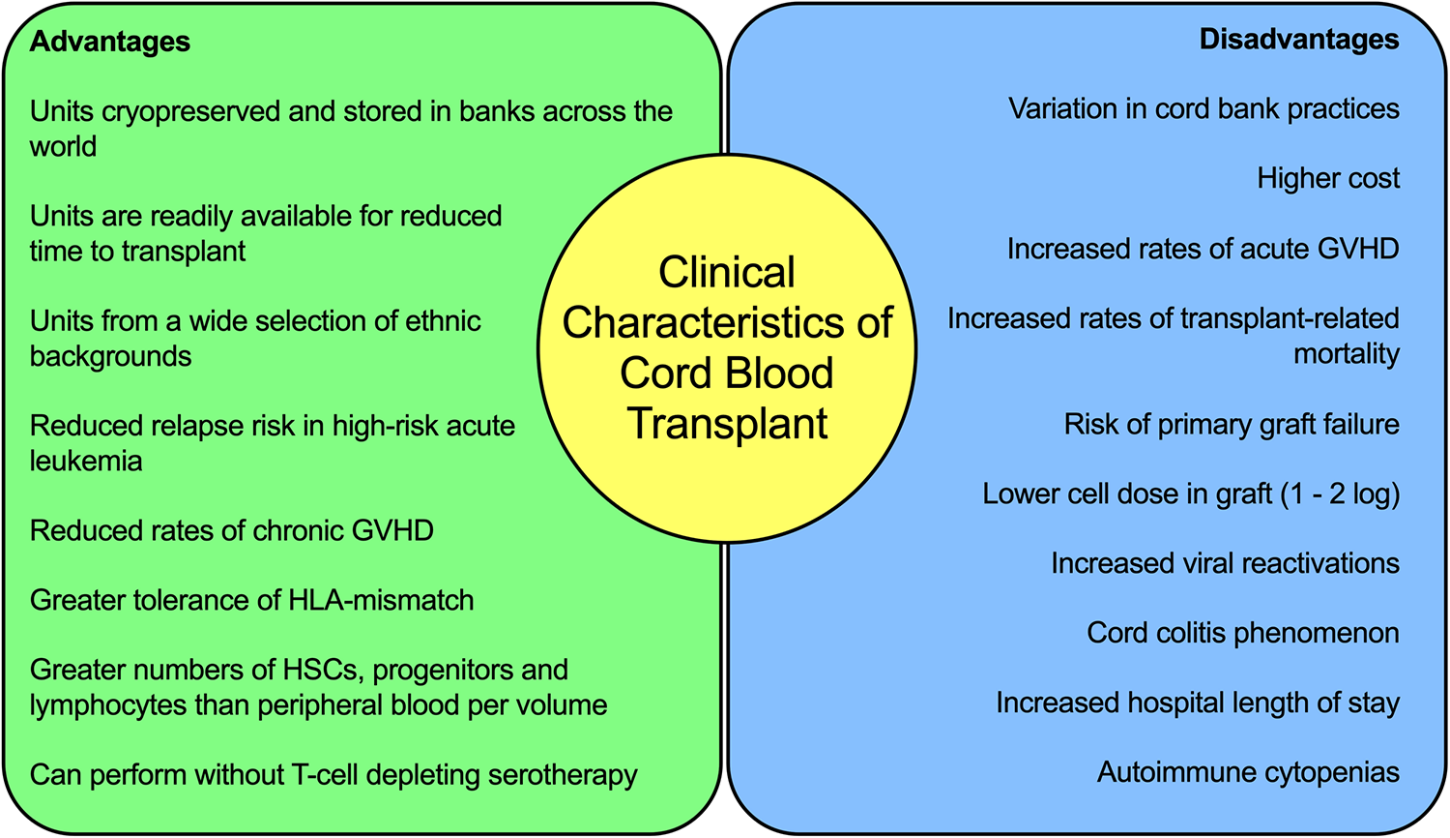
Summary of the clinical characteristics of cord blood transplantation for acute leukaemia.

The clinical characteristics of CBT for leukemia are summarized in Figure 1. These clinical findings suggest key differences in the immunology of cord blood, and in particular T-cell biology, when compared to adult peripheral blood or bone marrow. These differences are responsible for the observed improved relapse rates, greater tolerance of HLA-mismatch and reduced rates of chronic GVHD. It also highlights the importance of improving the understanding and application of supportive care for patients undergoing CBT, to reduce the burden of TRM and further improve survival outcomes.

## Clinical protocol for cord blood transplantation for pediatric acute leukemia

Our center is a large children's cord blood transplant center. In AML that is refractory to chemotherapy or relapsed, our first choice would be to select to use an unrelated cord blood donor without T-depleting serotherapy due to the reduced risk of relapse observed by our group and others in this high-risk cohort of patients (3, 4). We recognize the increased procedure-related risk with performing a mismatched unrelated CBT in comparison to a MUD transplant, but with higher risk myeloid leukemia the better associated disease outcomes overcome this consideration (90). Recent American Society for Transplantation and Cellular Therapy guidelines suggest that selection of less well-matched units could be considered for those patients with hematological malignancy (91).

We would consider refractory T-cell leukemia, mixed phenotype acute leukemia and refractory infant leukemia as similar to high-risk myeloid malignancy (90). Our conditioning regimen would typically include myeloablative busulfan if a first allogeneic transplant, without serotherapy. GVHD prophylaxis would include ciclosporin and mycophenolate mofetil, which we would aim to wean early in the absence of GVHD. Single cord blood unit selection follows a nationally defined protocol (92).

In children with relapsed leukemia after an earlier transplant procedure, we have used experimental procedures to augment GVL. This has included our use of granulocyte transfusions in conjunction with T-replete CBT to promote CD8^+^ T-cell expansion (93).

## Cord blood graft composition

Cord blood grafts are relatively enriched for hematopoietic stem cells (HSCs) when compared to BM and in particular PBSC grafts (94). Clinical cord blood units for transplant, however, usually contain 1–2 log lower cell dose than those obtained from peripheral blood or bone marrow donors so the actual number of transferred cells is usually smaller (95). Cord blood HSCs are of a more primitive phenotype with reduced CD38 expression (96). These stem cells have high capability for self-renewal and proliferation (96, 97). Cord blood also contains higher numbers of myeloid and lymphoid progenitors with high replication potential (98, 99). Analysis of lymphocyte subsets also shows higher absolute numbers of T, B and NK cells within a given volume of cord blood when compared to adult peripheral blood samples (100).

## Lymphocyte subsets within cord blood

Lymphocyte subsets within cord blood differ from those in adult peripheral blood both in number and phenotype. Within the CD3^+^ compartment of cord blood there is a higher proportion of CD4^+^ to CD8^+^ T-cells when compared to those in adult peripheral blood (100, 101). Cord blood T-cells consist predominantly of the naïve (CD45RA^+^/CD45RO^−^) phenotype, whilst adult peripheral blood is mainly comprised of memory (CD45RA^−/^CD45RO^+^) T-cells (100). Cytotoxic CD8^+^ T-cell populations are absent in cord blood with lower numbers of effector T-cells and those expressing activation markers such as HLA-DR (100). Whilst naïve T-cells are the most numerous subset in cord blood, some antigen-experienced T-cells are also present. The most notable of these includes those specific for maternal minor histocompatibility antigens (102).

Gamma delta (γδ) T-cells are a distinct subset of T lymphocytes defined by their expression of γδ T-cell receptors. They have effector capabilities and can promote inflammation (103). In peripheral blood, they make up only a small proportion of around 1%–5% of all T-cells with conventional αβ T-cells comprising the majority of all those in circulation (104). They are abundant at barrier sites. Absolute numbers of γδ T-cells are negligible in cord blood when compared to adult peripheral blood (100, 105, 106). Cord blood γδ T-cells are primarily of the Vδ1 subtype and have a naïve phenotype in comparison to Vγ9Vδ2 T-cells, which are the most numerous in adult peripheral blood (107, 108). The small number of Vγ9Vδ2 T-cells within cord blood are functionally immature (109). Cord blood γδ T-cells, however, have a highly diverse polyclonal repertoire (107, 108, 110). Receptor diversity is increasingly restricted with age and very limited in adult γδ T-cells (107, 108). *In vitro*, cord blood γδ T-cells readily expand and differentiate, becoming functionally cytotoxic (108). These characteristics in combination, mean that cord blood γδ T-cells are being further investigated for use in cancer immunotherapy (108).

Invariant Natural Killer T (iNKT) cells are a specialized subset of T-cells that crossover between innate and adaptive immunity (111). They are defined by their restricted TCR that can solely recognize lipid antigen presented by CD1d (111). Numbers remain stable throughout life from birth to adulthood but are highly variable between individuals (111, 112). They comprise a comparatively small proportion of all T-cells at approximately 0.1%–0.2% on average in both cord and peripheral blood. In murine HSCT models, adoptive transfer of iNKT cells can protect against GVHD, whilst preserving the GVL effect through inhibition of alloreactive T-cell expansion and activation and induction of donor regulatory T-cell expansion (113, 114). The scarcity of iNKT cells in peripheral blood compounds their utility in clinical applications, however. An early study of iNKT cells engineered from cord blood HSCs has shown promising pre-clinical results in amelioration of GVHD whilst preserving GVL (115).

NK cells are lymphocytes that form part of the innate immune system. They elicit anti-cancer effects through release of cytotoxic granules as well as activation of apoptotic pathways and can produce inflammatory cytokines (116). Cord blood grafts are relatively enriched for NK cells, which constitute around 20%–30% of the lymphocyte population in comparison to 10%–15% in peripheral blood (101). NK cells can be divided into two sub-populations, which are CD56^dim^CD16^bright^ and CD56^bright^CD16^dim^ (116, 117). These sub-populations have distinct phenotypic properties with CD56^dim^CD16^bright^ NK cells mediating cytotoxicity through granzyme B and perforin production and CD56^bright^CD16^dim^ NK cells mainly producing inflammatory cytokines such as IFNγ and TNFα (118). The predominant population of NK cells in cord blood are CD56^bright^CD16^dim^ with reduced cytotoxic capabilities and a more immature phenotype (119, 120). These cord blood NK cells have reduced expression of granzyme B and killer immunoglobulin-like receptors (KIR) and higher expression of the inhibitory receptor NKG2A (120, 121). Within cord blood, however, a distinct NK cell progenitor exists that can readily differentiate into functional, mature NK cells with the ability to produce IFN-γ, TNFα, IL-10, and GM-CSF and lyse cells *in vitro* (122). Cord blood NK cells also express higher amounts of CXCR4 suggesting superior bone marrow homing when compared to peripheral blood NK cells (120). Cord blood is a rich source of NK cells for use in immunotherapy, with encouraging early results (123).

B-cells are immunoglobulin producing lymphocytes that play a very important role in adaptive immunity. B-cells make up a larger proportion of the lymphocyte population in cord blood compared to peripheral blood, at around 15%–20% (101). Characteristics of B-cells differ between cord and peripheral adult blood. Cord blood contains a higher percentage of B-cell progenitors in comparison to adult and pediatric bone marrow (124). Most B-cells within cord blood do exhibit the same CD20^+^/CD5^−^ phenotype as B-cells found in adult peripheral blood (125, 126). There is, however, a distinct CD20^+^/CD5^+^ population of B-cells within cord blood (127). These CD5^+^ B-cells are specifically derived from fetal and neonatal progenitors and are characterized by IgM production that is polyreactive and indeed autoreactive with low affinity binding (126, 128). Additionally, cord blood B-cells produce accelerated responses to stimulation with distinct transcriptional pathways (125).

## Cord T-cell biology

Cord blood and adult peripheral blood T-cells have different functional properties, which are summarized in Table 2.

**Table 2:** Cord blood T-cell biology differences to adult peripheral blood T-cells.

Phenotype
– Greater proportion of CD4^+^ T-cells compared to CD8^+^ T-cells (100)
– Predominance of naïve phenotype (100)
– Larger proportion of recent thymic emigrants (129)
– Distinct T-cell lineage (130, 131)
Increased proliferative abilities and transformation into effector T-cells
– Increased proliferation in response to anti-CD3 and anti-CD28 co-stimulation (132)
– Increased proliferative abilities in response to cytokine stimulation (129, 133, 134)
– Increased proliferation in response to APCs (135)
– Greater Ki-67 proliferation marker expression (129, 130)
– Rapid transformation from naïve to effector phenotype in response to stimulation (132, 136, 137)
– Enhanced TCR signaling through AP-1 and MAPK pathway (135)
Reduced cytokine production, activation, and cytotoxic capabilities
– Lack of constitutive perforin expression (142)
– Impaired Fas-ligand mediated cytotoxicity (143)
– Impaired cytokine production on stimulation (136, 138, 144–146)
– Impaired IFN-γ production on stimulation with CMV viral peptide (147)
– Reduced expression of cell surface activation markers CD25 and CD154 in response to dual CD3 and CD28 co-stimulation (143)
– Lower levels of activation markers on CD8^+^ T-cells (100)
Tregs
– Increased IL-10 production (138, 139)
– Increased proliferative abilities of Tregs (139)
– Potent suppressor function of Tregs (139–141)
– Increased telomerase expression (129)
– Reduced CLA expression (100)
Reduced alloreactivity
– Reduced alloreactivity, less cytotoxicity in response to allo-antigen (134)
– Reduced expression of NFATc2 genes to amplify allogeneic responses in CD4^+^ T-cells (148)
– Increased susceptibility to apoptosis in response to allogeneic stimuli (149)

APC, antigen presenting cell; CLA, cutaneous lymphocyte antigen; CMV, cytomegalovirus; IL, interleukin; IFN-γ, interferon-gamma; MAPK, mitogen-activated protein kinases; NFAT, nuclear factor of activated T-cells; TCR, T-cell receptor; Treg, T-regulatory cell.

*In vitro* studies show that cord blood T-cells have a greatly increased capacity for proliferation in response to multiple stimuli and lymphopenia. Cord blood T-cells proliferate more rapidly than those in adult peripheral blood in response to cytokine stimulation (129, 133, 134). In assays analyzing these responses, IL-7 produced greater proliferation in cord blood T-cells, particularly the CD4^+^ population whereas IL-15 stimulated the greatest proliferation in cord blood CD8^+^ T-cells (129). Clinically in BMT, low serum levels of IL-15 are associated with an increased risk of post-transplant relapse in AML indicating its potential role in the GVL effect, perhaps through induction of CD8^+^ T-cell responses (150). CD8^+^ T-cells in neonates, also exhibit an intrinsic ability to rapidly proliferate in response to in response to CD3 and CD28 co-stimulation (130, 132). Cord blood CD4^+^ T-cells proliferate more than adult peripheral blood T-cells when stimulated with self-APCs (135). This occurs in conjunction with enhanced TCR signaling and upregulation of the MAPK signaling cell-cycle pathway (135). This greater proliferative potential is thought to arise, in part, secondary to distinct lineages of fetal and adult progenitor cells and a transcriptional program geared towards lymphopenia-induced proliferation (130, 131). In xenograft studies, greater levels of expression of the proliferation marker Ki-67 is also seen in both cord blood CD4^+^ and CD8^+^ T-cells, in comparison to those in adult peripheral blood. This indicates that there is also a higher proportion of proliferating cord blood T-cells *in vivo* (129, 130). Cord blood CD8^+^ T-cells in particular have higher levels of Ki-67 expression (129). Additionally, spontaneous telomerase expression occurs in cord blood T-cells to allow proliferation without telomere shortening, aiding longevity of the T-cell progeny (129).

In response to antigenic stimulation, fetal CD8^+^ T-cells preferentially become terminally differentiated, short lived effectors rather than memory T-cells as seen in adults (130). Naïve CD4^+^ T-cells in the newborn expressing CD45RA are also able to much more rapidly transform to CD45RO effector memory phenotype on stimulation when compared to naïve adult peripheral blood CD4^+^ T-cells (136).

CD4^+^ helper T-cells Th1 and Th2 are broadly defined by their ability to produce IFNγ/TNFα and IL-4/IL-5/IL-13 as well as the transcription factors T-bet and GATA-3, respectively. Cord blood T-cells produce less inflammatory cytokines overall in response to stimulation when compared to adult peripheral blood (129, 144, 145). Both CD4^+^ and CD8^+^ subsets produce less IFNγ and TNFα as well as lower levels of IL-2 and IL-4 (136, 144, 145). This includes in response to CMV peptide stimulation (147). Reduced expression of the transcription factor nuclear factor of activated T-cells c2 (NFATc2), which upregulates the expression of many cytokines involved in T-cell inflammatory responses, has been hypothesized as a mechanism for this observation (148). Low production of IFNγ specifically by CD4^+^ T-cells in the neonate, has been associated with hypermethylation of the IFNγ promoter (151). Neonatal naïve T-cells that have been allowed to mature *in vitro*, however, acquire the ability to secrete IFNγ and IL-2 on secondary stimulation (136).

Cord blood T-cells display less alloreactivity when compared to peripheral blood T-cells, which may contribute to the reduced rates of GVHD seen clinically (134). Cord blood T-cells more readily undergo apoptosis in response to alloantigen (143, 149). Levels of constitutive perforin expression in cord blood CD8^+^ T-cells are low in comparison to those in adult peripheral blood, and cytotoxicity mediated through the FAS-ligand pathway is impaired (142, 143). Cord blood T-cells co-cultured with immature adult dendritic cells produced greater IL-10 and reduced IFNγ than adult T-cells, which is a regulatory cytokine profile (138). Cutaneous lymphoid antigen (CLA) is expressed on T-cells involved in migration to areas of skin inflammation. There is no expression of CLA on cord blood T-cells, which could be significant in the lower incidence of chronic skin GVHD with CBT (100).

Cord blood is a rich source of CD4^+^/CD25^+^/FoxP3^+^ regulatory T-cells (Tregs), which are inherently programmed to immune tolerance with low levels of immunological memory (140). Cord blood Tregs have strong suppressive capabilities against alloreactive T-cells with high levels of IL-10 production *in vitro,* following activation and expansion (139, 141). This suppressive effect is potent and occurs consistently, inhibiting production of T-cell activation-dependent cytokines such as IL-2, IFN-γ, TNFα and GM-CSF in mixed lymphocyte reaction assays (141). In comparison to Tregs isolated from peripheral blood, cord blood Tregs also have much greater capacity for expansion with higher Ki-67 expression and a gene expression profile that favors proliferation and chromatin modification (139, 152). Cord blood Tregs also express higher levels of the chemokines CCR9 and CCR7 than their peripheral blood counterparts, which regulate trafficking to the gut and lymph nodes respectively (152). The potential of cord-derived Tregs to prevent GVHD whilst preserving the GVL effect has been established in xenograft models (153). Further work has optimized the purification and expansion process to allow for use in clinical settings (154, 155). There are promising results from early clinical trials using cord-derived Tregs as preventative therapy for GVHD with reduced rates of acute and chronic GVHD without an increase in relapse (154, 156).

## Cord blood antitumor effects in xenograft models

The superior clinical outcomes in malignancy have been further supported in pre-clinical studies, investigating cord blood T-cells as the primary mediators of enhanced antitumor activity. In xenograft models, very successful antitumor effects of cord blood T-cells have been demonstrated (137, 157). In one study, cord blood mononuclear cells induced dramatic remission in mice with lung and cervical cancers, with high levels of tumor infiltration by CD3^+^ T-cells shown (157). Further *in vitro* assays demonstrated tumor specific antigen cytotoxicity. Comparative analysis of cord blood and peripheral blood T-cells, also shows that cord blood T-cells produce a greatly superior antitumor response (137). In an Epstein-Barr virus (EBV)-driven human B-cell lymphoblastoid tumor mouse model, cord blood T-cells had enhanced antitumor effect with rapid infiltration and induction of remission. Cord blood T-cells expressed enhanced levels of the tumor-homing receptor CCR7. In contrast, peripheral blood T-cells were slower to infiltrate and had reduced antitumor activity with tumor progression. Analysis of the tumor infiltrating cord blood lymphocytes showed them to be primarily CD8^+^ T-cells that had converted from naïve to central and effector memory phenotype. Importantly, these cord blood T-cells were able to mediate a greatly augmented GVL effect without exerting xenoreactivity, mirroring the clinical picture seen with CBT. Effector memory CD4^+^ T-cells have also been shown to exert effective GVL effects without GVHD in the mismatched HSCT setting in mouse models of chronic myelogenous leukemia (158).

In summary, T-cells contained within a cord blood graft are a distinct entity from those derived from peripheral blood and bone marrow. They are predominantly CD4^+^ T-cells and both CD4^+^ and CD8^+^ T-cells are naïve (100). These subsets possess the ability, however, to quickly transform into effector T-cells with cytotoxic potential (132, 137). Both CD4^+^ and CD8^+^ cord blood T-cells are capable of rapid proliferation in response to lymphopenia, cytokines and APCs, more so than adult T-cells. There is some suggestion, however, that there is impairment in the production of inflammatory cytokines and mediation of cytotoxicity through certain cellular pathways (129, 143). There is, therefore, much more to be understood about how an enhanced GVL effect is mediated during cord blood transplantation. Cord blood CD8^+^ T-cells are endowed with enhanced tumor-homing capabilities due to high levels of CCR7 expression, which in conjunction with greater proliferation may contribute to the superior GVL effect (135, 137). Additionally, the primitive nature of cord blood itself could potentially reduce susceptibility to inhibitory co-signaling and induction of an exhausted T-cell state (130, 143). There are also differences in clinical characteristics of CBT including greater use of HLA-mismatch and omission of T-cell depleting serotherapy, which likely contribute to the phenomenon seen.

## Mechanism of T-cell reconstitution following HSCT

Following transplant conditioning there is a period of profound lymphopenia and aplasia. T-cell reconstitution occurs through two separate pathways. In the first, there is thymus-independent peripheral expansion of T-cells transplanted within the graft or of residual recipient T-cells that have escaped transplant conditioning, driven by exposure to antigen and cytokines. This is the pathway that predominates in the early post-transplant phase (159). Distinct from this, restoration of thymopoiesis from hematopoietic progenitors occurs in parallel but at a much slower rate. This thymus-dependent pathway utilizes progenitors transferred within the graft or those derived from donor hematopoietic stem cells to produce naïve T-cells with a greater TCR repertoire, thus promoting longevity of T-cell reconstitution with increased functionality (81, 160).

Within a lymphopenic environment, homeostatic proliferation of naïve T-cells occurs during which they acquire the phenotype and functional properties of effector T-cells (161, 162). Interaction of cells with peptide bound to MHC molecules and IL-7 is essential to the survival and expansion of naïve CD4^+^ and CD8^+^ T-cell populations (159, 163). With lymphopenia there is reduced competition for APCs and cytokines allowing greater expansion to occur.

Reliance on the thymus-independent pathway alone for T-cell reconstitution can result in a reduced TCR repertoire that is skewed and oligoclonal (147). Restoration of thymopoiesis is required for the formulation of *de novo* naïve T-cells following HSCT. This is necessary to restore a complete and long-lasting T-cell compartment capable of responding to a wide range of antigens (147, 159, 164). Thymopoiesis is the process through which progenitors derived from HSCs in the bone marrow (or in the context of transplant, infused with the graft) proliferate and mature within the thymus and become committed to the T lineage. It is in this manner that TCR specificity is generated (159). One technique to assess reestablishment of thymopoiesis, is detection of T-cell receptor rearrangement excision circles (TRECs), which are extra-chromosomal fragments of DNA that are generated as by-products of the TCR gene rearrangement process within the thymus, and can be used to identify recent thymic emigrants (RTEs) (164). They exist stably within the cytoplasm but are not replicated during cell division, which means they can be measured to quantify functionality of the thymus (159).

## T-cell reconstitution after cord blood transplantation

Cord blood T-cell reconstitution is CD4^+^ biased, which differs from BM and PBSC cell sources in which CD8^+^ T-cells predominate (135, 165–170). Cord blood transplant without T-cell depleting serotherapy has been shown to result in rapid CD4^+^ reconstitution in both adults and children (135, 165, 171). This is initially attained through the thymus-independent pathway based on the low number of detectable TRECs and may be secondary to the increased proliferative capabilities of cord blood CD4^+^ T-cells in lymphopenic environments (135). Successful CD8^+^ T-cell reconstitution can be slower in cord blood transplant recipients than BM and PBSCs (166, 171–173). The most prevalent early reconstituting CD4^+^ and CD8^+^ cord blood T-cells are effector memory, with an increase in naïve T-cells occurring from 6 to 9 months suggesting thymus-dependent recovery at this time point (171). Further phenotypic analysis of early reconstituting CD8^+^ T-cells in CBT suggests significantly greater proportions of highly activated effector memory cells expressing CD38 than in PBSCT recipients (173). Marked CD8^+^ T-cell expansion can occur with CMV reactivation following CBT (171). A polyclonal TCR repertoire with normal spectratyping has been observed early in some patients within the first month post-CBT without T-cell depleting serotherapy (165). Conversely, delayed restoration of TCR diversity has been reported in the ATG setting (81).

There is efficient recovery of thymopoiesis seen with cord blood transplant in the T-replete setting with TRECs detectable within the first 3 months post-transplant and reaching normal levels within 6 months (165, 174). This is initially comparable to haploidentical and MSD transplants but is in fact superior in cord blood at 2-years post-transplant with higher TREC numbers and greater TCR repertoire indicating efficient regeneration of thymic function with CBT (81, 174). This occurs despite the much lower cell dose contained within cord blood units than BM or PBSC grafts. Higher numbers of lymphomyeloid progenitors (LMPs) contained within cord blood grafts may contribute to this finding, but it may also suggest that cord blood LMPs themselves are superior in their ability to reconstitute thymopoiesis in comparison to those in other graft sources (160). With use of ATG serotherapy, however, CD4^+^ reconstitution following cord blood transplant is significantly delayed (147, 166, 168, 175, 176). ATG serum concentration modelling during HSCT shows cord blood CD4^+^ T-cells are particularly susceptible to ATG exposure even at low levels (62, 63, 177). Further research is warranted to define when polarization into CD4^+^ T helper cell subsets occurs following CBT and how this may differ from other stem cell sources.

Sustained CD4^+^ reconstitution has been associated with improved OS children with leukemia, independent of cell source (178). In CBT specifically, CD4^+^ T-cell reconstitution has been positively associated with improved overall and leukemia-free survival in children and adults (62, 171). Early post-transplant, higher numbers of cytotoxic, effector CD8^+^ T-cells are also associated with improved OS and reduced NRM (40).

## NK cell and B-cell reconstitution after cord blood transplantation

NK cells are the first lymphocyte to engraft following CBT, achieving normal levels within the first month (166, 175, 179, 180). In the post-transplant period, T-cell lymphopenia is associated with a compensatory increase in NK cells to above physiological levels (147). NK cell reconstitution occurs more rapidly with CBT than PBSC or BM cell sources (167, 178, 179, 181). Despite their initially immature phenotype, NK cells can quickly attain their innate cytolytic abilities following CBT meaning they are crucial in exerting a GVL effect in the early post-transplant period for acute leukemia (116, 167). NK cells in CBT recipients show significantly higher expression of activation markers CD69 and NKP30 in the first few weeks, than those receiving PBSCs (173).

Significant B-cell recovery starts around 3–4 months after HSCT reaching normal levels by 6–12 months (168, 182). Adequate immunoglobulin production, however, can take several years to be achieved (183). B-cell recovery after CBT is comparatively enhanced and occurs much earlier than with BMT (166, 167, 178, 184). Similarly to NK cells, B-cell numbers have also been seen to reach higher than physiological levels following CBT, perhaps as a compensatory measure for T-cell lymphopenia (147). Immunoglobulin levels in response to commonly encountered antigens approach normal levels within the first year of CBT (95, 170). In adults, IgM recovery is comparable between dCBT and PBSCT recipients. Normal levels of IgG, however, occur at 5–6 months following dCBT, which is not seen at 12 months following PBSCT (185). Higher numbers of naïve B-cells expressing CD127 are seen with CBT recipients (172). Stromal cells contribute to B-cell development and therefore the greater numbers of mesenchymal stem cells found in in cord blood might contribute to this quick recovery and early functionality (96, 186).

## Future directions to augment GVL effect with cord blood transplantation

We have established that T-cells are important mediators in GVL and that CBT is associated with reduced relapse rates, secondary to the superior antitumor properties of cord blood T-cells. The exact mechanism of this is yet to be elucidated, but may involve a combination of factors including the differing composition of cord blood grafts with predominance of naïve T-cells; the innate biology of cord blood T-cells themselves, particularly their ability to proliferate and transform into short-lived effectors; their greater tolerance of HLA-mismatch, allowing this to be utilized in conjunction with omission of T-cell depleting serotherapy in conditioning regimens; as well as the differing pattern of immune reconstitution following CBT, with earlier restoration of thymopoiesis due to greater numbers of LMPs. Therefore, future directions must look at further defining and augmenting the GVL effect of CBT.

Our group previously described significant T-cell expansion driven by granulocyte transfusions in conjunction with CBT (187). This expansion was predominantly of CD8^+^ T-cells and importantly was associated with prolonged remission in patients with acute leukemia. On the basis of these findings, we have conducted an early phase I/II study in which we have trialed the use of third-party, pooled granulocyte transfusions in conjunction with CBT to promote CD8^+^ expansion in children with high-risk myeloid malignancy (93). We have reported significant and reproducible T-cell expansion occurring in association with a cytokine release syndrome, in all patients excluding one with primary graft failure. Expanded T-cells were CD8^+^ and effector memory or terminally differentiated effector memory (TEMRA) phenotype and exhibited canonical markers of activation and cytotoxicity. The phenotype of expanded T-cells appears to be similar to that of the highly effective tumor-eradicating T-cells from xenograft models (137). In this high-risk cohort of patients with MRD positive acute leukemia, 90% of patients achieved hematological remission, 80% became MRD negative and 50% of patients are alive and in disease remission with over one year median follow up (93).

Further research into the mechanisms of GVL are necessary to better understand and advance this field. We speculate that cord blood T-cells are biologically distinct from adult T-cells, and that the observed improved GVL without chronic GVHD likely reflects this distinct biology. Understanding this relationship between T-cell biology and clinical outcomes might allow the T-cell responses we have reported, to be improved further yet or re-directed. Specifically identifying targets of cord blood T-cells, separating this entity from GVHD and understanding the role of HLA-mismatch, will mean that T-cell techniques can be honed, and potentially improve patient outcomes.

Universal or “off-the shelf”, allogeneic CAR T-cells are also being trialed in numerous studies, in order to overcome the limitations of using autologous CAR T-cells, which rely upon the functionality of the recipient T-cell pool and can have prolonged manufacture time with high costs (188). Cord blood T-cells could be ideal candidates for this technology due to their naïve phenotype, increased replicative capabilities and reduced alloreactivity (188, 189).

The lack of availability of DLI with CBT has previously been seen as a disadvantage, but with newer methods of T-cell separation, cryopreservation and cord unit expansion, theoretically, cord blood T-cells could be stored for DLI in the future (190, 191). Improvements in supportive care and utilization of targeted or less toxic conditioning regimens could dramatically reduce the burden of TRM associated with CBT. The advent of new treatments for acute GVHD, which may include cord blood-derived Tregs, and anti-viral drugs such as letermovir will be crucial in these efforts (59, 154).

## Conclusion

In conclusion, cord blood transplantation offers an enhanced GVL effect that has shown to be particularly effective in difficult-to-treat leukemia, without the risk of chronic GVHD. This effect can be further enhanced through amelioration of the effects of TRM and trials to appropriately risk stratify patients. Cord blood T-cells are a phenotypically distinct entity from those in peripheral adult blood or bone marrow, but the exact mechanisms through which they elicit superior antitumor effects remain to be elucidated. Further research is required but cord blood offers exciting opportunities in the field of cellular immunology and the landscape of CBT is likely to rapidly expand and evolve in coming years, giving rise to novel and innovative treatment modalities.
